# Sero-prevalence of syphilis infection among people living with HIV in Sierra Leone: a cross-sectional nationwide hospital-based study

**DOI:** 10.1186/s12879-023-08740-9

**Published:** 2023-11-06

**Authors:** Darlinda F. Jiba, Sulaiman Lakoh, Shuchao Wang, Wei Sun, Umu Barrie, Matilda N. Kamara, Abdulai Tejan Jalloh, Francis K. Tamba, George A. Yendewa, Jin-Wen Song, Guang Yang

**Affiliations:** 1 Ministry of Health and Sanitation, Government of Sierra Leone, Freetown, Sierra Leone; 2 College of Medicine and Allied Health Sciences, University of Sierra Leone, Freetown, Sierra Leone; 3 Sustainable Health Systems Sierra Leone, Freetown, Sierra Leone; 4 Changchun Veterinary Institute, Chinese Academy of Agricultural Sciences, Changchun, China; 5 School of Public Health, Southern Medical University, Guangzhou, China; 6 Infectious Disease Research Network, Freetown, Sierra Leone; 7 Department of Medicine, Case Western Reserve University School of Medicine, Cleveland, OH USA; 8 Senior Department of Infectious Diseases, the Fifth Medical Centre of PLA General Hospital, Beijing, China; 9 Department of Clinical Laboratory, the Fifth Medical Center of PLA General Hospital, Beijing, China

**Keywords:** Syphilis, People living with HIV, Seropositivity, Syphilis screening, Freetown, Sierra Leone

## Abstract

**Background:**

Globally, there were an estimated 7.1 million new syphilis infections in 2020, with more than 30% of these new infections reported in African countries such as Sierra Leone. Despite this, there is no HIV-specific syphilis screening program in Sierra Leone. Thus, data are needed to inform public health practice. In this study, we aimed to determine the prevalence of syphilis seropositivity and factors associated with syphilis seropositivity among people living with HIV (PLHIV).

**Methods:**

A cross-sectional study was conducted at 10 health facilities in Sierra Leone, among adults with HIV, aged 18 years or older, from September 2022 to January 2023. Parameters of interest were collected including age, sex, marriage, antiretroviral therapy (ART) regimen, HIV viral load, duration of ART treatment, and hospital level of care. The syphilis antibody was detected by a rapid test based on immunochromatography assay. Data were analyzed using R-software version 4.2.3 (R Core Team, Vienna, Austria). Pearson’s χ^2^ test, Fisher’s exact test and Kruskal–Wallis H test were applied to assess the differences in syphilis seropositivity between groups as appropriate. Univariate logistic regression and multivariate logistic regression analysis was used to assess factors associated with syphilis seropositivity. The level of statistical significance was set at *P* < 0.05.

**Results:**

Of the 3082 PLHIV individuals in our study, 2294 (74.4%) were female and 2867 (93.0%) were receiving ART. With a median age of 36 years, 211 (6.8%, 95% CI 6.0–7.7) were positive for syphilis. The prevalence of syphilis was highest in people aged 60 years and over (21.1%, 95%CI 14.7–29.2), followed by people aged 50–60 years (15.5%, 95%CI 11.9–19.9) and in the widowed population (11.9%, 95%CI 8.9–15.8). There were no differences in syphilis seropositivity between gender, ART status, ART regimen, duration of ART, HIV viral load and hospital level of care. Older age (50–60 years: adjusted OR 3.49, 95%CI 2.09–5.85 *P* < 0.001; 60–100 years: adjusted OR 4.28, 95%CI 2.21–8.17, *P* < 0.001) was an independent predictor of seropositive syphilis.

**Conclusions:**

We observed a high prevalence of syphilis among PLHIV. Older people and widowed population have higher syphilis seropositivity. Older age was an independent predictor of syphilis positivity. Therefore, we call for the integration of syphilis screening, treatment and prevention in HIV services.

## Background

Globally, an estimated 7.1 million new syphilis infections were reported in 2020 and the African region accounts for more than 30% of these new infections [1]. Syphilis are sexually transmitted infections (STIs) caused by *Treponema pallidum,* but can also be transmitted vertically, leading to adverse birth outcomes in exposed infants [1, 2]. Furthermore, syphilis is the second leading cause of preventable stillbirths worldwide and can lead to high morbidity and mortality in adults, including neurologic complications [1, 2]. In the last decade, there is a resurgence of syphilis in many parts of the world [3].

Despite the lack of global and regional data on the burden of syphilis seropositivity in people living with HIV (PLHIV), due to shared transmission routes, syphilis is one of the common coinfections in this population [3, 4]. Given these challenges, the World Health Organization (WHO) has called for the triple elimination of HIV, viral hepatitis B, and syphilis [5–7]. However, the global effort to eliminate syphilis as a major public health problem by 2030 is hampered by several challenges facing the health system in low-income countries [7]. Consequently, many studies in low-income countries have shown high rates of syphilis among PLHIV, reinforcing the need for robust syphilis screening programs in sub-Saharan Africa [8–12].

Sierra Leone has 1.7% of its population living with HIV [13]. But there are no national estimates for other STIs, including syphilis. However, data extrapolated from antenatal clinics reported a prevalence of 0.9% and estimated over 2,000 cases of congenital syphilis [14]. Studies of blood donors and military personnel have reported prevalence rates of 0.8 and 7.3%, respectively [15, 16]. Older age, HIV positivity and residence in rural settings were risk factors associated with seropositivity for syphilis in these populations [15, 16]. Nonetheless, a PubMed search on May 31, 2023 reported only one single-center study on the prevalence of syphilis in PLHIV in Sierra Leone [17]. As a low-income country with enormous health challenges and high STI risk behaviors, it is expected that the impact of syphilis on PLHIV in Sierra Leone may be more severe than reported [14, 18]. Despite these gaps in the response against syphilis and the availability of guidelines for syphilis treatment and prevention, its prevention and control has not improved in Sierra Leone. Based on these challenges, the Sierra Leone National AIDS Control Program introduced routine syphilis screening of pregnant women using a dual test kit, although coverage was limited, reaching only 50% of pregnant women [14]. Sierra Leone does not have a PLHIV-specific syphilis screening program despite recommendations from international guidelines [7]. Thus, data are needed to inform public practices. In this study, we aimed to determine the prevalence of syphilis seropositivity and factors associated with syphilis seropositivity in PLHIV in 10 public hospitals in Sierra Leone.

## Materials and methods

### Study design and setting

A cross-sectional study was conducted at 10 health facilities in Sierra Leone, including two primary hospitals (Jenner Wright Clinic and Waterloo Community Health Center), two secondary hospitals (Rokupa and Lumley Government hospitals), and six tertiary/regional hospitals (Connaught Hospital, Kenema Government Hospital, Makeni Government Hospital, Bo Government Hospital, Princess Christian Maternity Hospital and 34 Military Hospital).

Sierra Leone is divided into five geographic regions. The Western Area is the most populous region and includes Freetown (the capital). Of a population of 7 million, 22% (1.5 million) reside in the Western Area [19]. About 42% of the 10,350 healthcare workers employed by the government are in the Western Area [20]. Sierra Leone's public health system is divided into three levels of care, such as primary, secondary and tertiary care. The national and regional hospitals provide tertiary care [20]. There are 25 public hospitals in Sierra Leone, 10 of which provide tertiary services.

### Study population and sampling technique

**Fig. 1:**
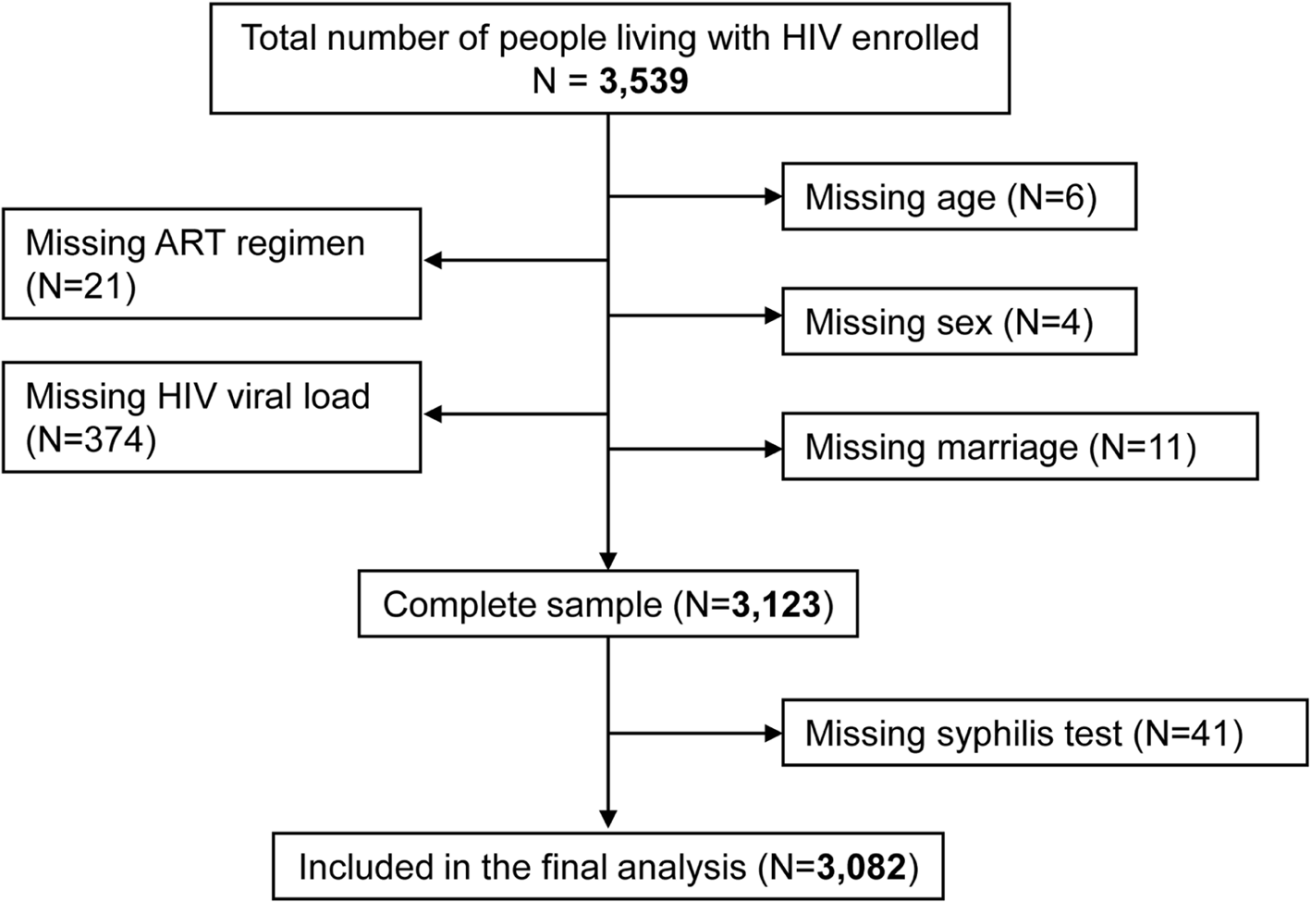
Study participant flow diagram Exclusion criteria for the selection of samples with complete data

Adult HIV patients aged 18 years or older were sequentially recruited into the study. We excluded patients who refused consent or were in these facilities for a temporary pick up of their antiretroviral drugs. Between September 2022 and January 2023, we recruited a total of 3539 PLHIV into the study, but patients with missing data were excluded from the analysis. Consequently, details of only 3082 PLHIV were included in the final analysis. (Fig. 1).

### Laboratory procedure

Approximately 2–4 mL of venous blood samples were aseptically collected into sterile EDTA vacutainer tubes and analyzed at the Infectious Disease Prevention Center of the 34 Military Hospital. Plasma was separated by centrifugation at 2000 rpm for 10 min. Wondfo one step Syphilis Test Cassette (Wondfo, Guangzhou, China), a rapid test based on immunochromatography assay, was used to detect antibodies of Treponema Pallidum according to the manufacturer’s instructions. The test kit was CE (European Conformity) certified, and the sensitivity and specificity were 100% and 98%, respectively.

### Definitions and measures

We define viral suppression as HIV RNA < 1000 copies per mL and unsuppressed viral load as HIV RNA ≥ 1000 copies per mL. ‘’Previously married women’ are PLHIV who were married, but are now divorced, widowed, or separated. PLHIV who are taking antiretroviral drugs are said to be currently on treatment, regardless of duration.

### Data collection, management and statistical analysis

Demographic and HIV related information, including age, sex, marriage, ART regimen, HIV viral load, duration of ART treatment, and hospital level of care, were collected through paper questionnaires containing de-identified variables by trained research assistants. The collected information was entered into a password-protected Microsoft Excel sheet, accessible only to the research team.

Data were cleaned, coded and transferred to R-software version 4.2.3 (R Core Team, Vienna, Austria) for analysis. Demographic characteristics were presented as the number (%). Pearson’s χ^2^ test, Fisher’s exact test and Kruskal–Wallis H test were applied to assess the differences in syphilis seropositivity between groups as appropriate. Parameters of interest were antiretroviral therapy (ART), HIV viral load, duration of treatment, syphilis seropositivity, hospital level of care, marital status and age. Univariate and multivariable logistic regression analyses were used to assess factors associated with syphilis seropositivity. The multivariate logistic regression analysis adjusted for age, sex, marriage, ART regimen, duration of ART, HIV viral load and type of hospital. All statistical tests were two sided, and the level of statistical significance was set at *P* < 0.05.

## Results

### Demographic and HIV characteristics of enrolled individuals

A total of 3082 PLHIV were enrolled in our study. The demographic information is shown in Table 1. The median age was 36 years. Patients aged under 30 years and 30–40 years accounted for 32.9% and 30.7%, respectively. Most of the participants were female (74.4%), 1430 (46.4%) were married and 2867 (93.0%) were receiving ART. HIV viral load was unsuppressed in 531 (17.2%) PLHIV.

**Table 1:** Sociodemographic characteristics and syphilis seroprevalence among enrolled individuals in this study

Characteristics	Participants tested (n, %)	Positive (n, %)	95% CI	*p*-value
Overall	3082 (100)	211 (6.8)	6.0–7.7	
**Age (years)**				< 0.001
(0,30]	1,013 (32.9)	47 (4.6)	3.5–6.2	
(30,40]	947 (30.7)	39 (4.1)	3.0–5.6	
(40,50]	647 (20.9)	44 (6.8)	5.0–9.1	
(50,60]	342 (11.1)	53 (15.5)	11.9–19.9	
(60,100]	133 (4.3)	28 (21.1)	14.7–29.2	
**Sex**				0.6
Female	2,294 (74.4)	154 (6.7)	5.7–7.8	
Male	788 (25.6)	57 (7.2)	5.6–9.3	
**Marriage**				< 0.001
Divorced	58 (1.9)	2 (3.4)	0.6–13.0	
Married	1,430 (46.4)	106 (7.4)	6.1–8.9	
Separated	94 (3.0)	7 (7.4)	3.3–15.2	
Single	1,131(36.7)	52 (4.6)	3.5–6.0	
Widowed	369 (12.0)	44 (11.9)	8. 9–15.8	
**ART**				0.7
no	215 (7.0)	16 (7.4%)	4.5–12.0	
yes	2,867 (93.0)	195 (6.8%)	5.9–7.8	
**ART regimen**				0.12
TLE	685 (22.2)	39 (5.7%)	4.1–7.8	
TLD	1,894 (61.5)	144 (7.6%)	6.5–8.9	
Others	65 (2.1)	1 (1.5%)	0.1–9.4	
Unknown	223 (7.2)	11 (4.9%)	2.6–8.9	
**ART duration (months)**				0.13
0 ~ 6	415 (15.0)	21 (5.1%)	3.2–7.8	
6 ~ 12	223 (8.2)	12 (5.4%)	2.9–9.4	
12 ~ 24	359 (13.0)	22 (6.1%)	4.0–9.3	
24 ~	1,722 (63.0)	135 (7.8%)	6.6–9.2	
**HIV viral load (copies/mL)**				0.3
< 1000	2,551 (82.8)	180 (7.1)	6.1–8.1	
≥ 1000	531 (17.2)	31 (5.8)	4.1–8.3	
**Hospital**				0.7
primary	656 (21.3)	47 (7.2)	5.4–9.5	
secondary	200 (6.5)	16 (8.0)	4.8–12.9	
tertiary	2,226 (72.2)	148 (6.6)	5.7–7.8	

Pearson’s χ2 test, Fisher’s exact test and Kruskal–Wallis H test were applied to assess the differences in syphilis seropositivity between groups as appropriate. A two-tailed *P*-value less than 0.05 was deemed to be statistically significant

*ART* antiretroviral therapy, *CI* Confidence Interval, % indicates proportion of participants that fell within each category. *TLD* tenofovir + lamivudine + dolutegravir, *TLE* tenofovir + lamivudine + efavirenz

### Seroprevalence of syphilis among PLHIV

**Fig. 2:**
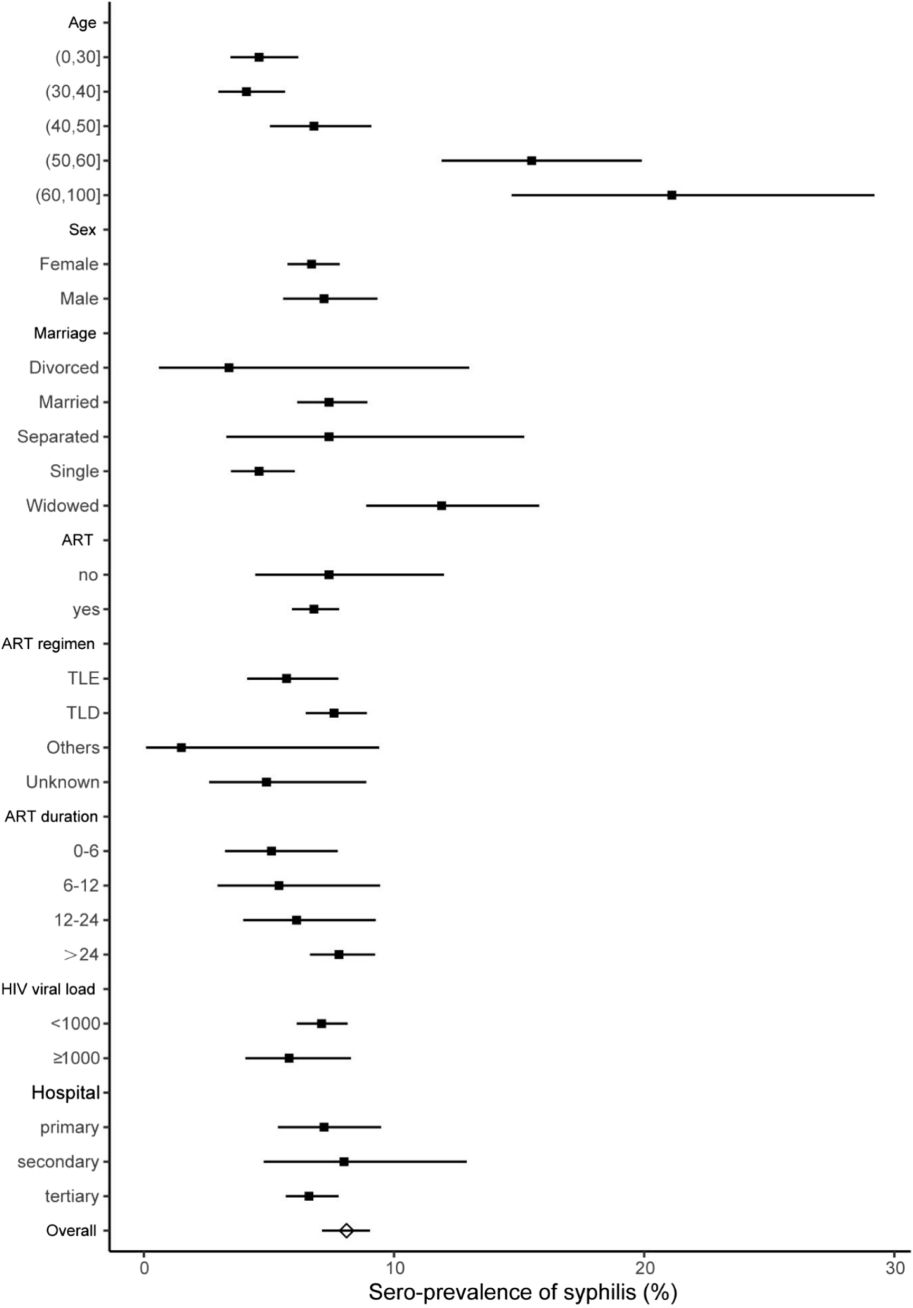
Syphilis seroprevalence for the combined measure by demographic variables in people living with HIV

Of the 3082 PLHIV screened for syphilis, 211 (6.8%, 95%CI 6.0–7.7) were positive (Fig. 2 and Table 1). The prevalence of syphilis is highest in people aged 60 and over (21.1%, 95%CI 14.7–29.2), followed by people aged 50–60 (15.5%, 95%CI 11.9–19.9). Syphilis prevalence was higher in the widowed population (11.9%, 95%CI 8.9–15.8), but there were no differences in gender, ART status, ART regimen, duration of ART, HIV viral load and level of hospital.

### Factors associated with the positivity of syphilis

Older age (50–60 years: OR 3.77, 95%CI 2.49—5.72 *P* < 0.001; 60–100 years: OR 5.48, 95%CI 3.26—9.07, *P* < 0.001) was significant factors for syphilis seropositivity in univariate logistic regression analysis. On multivariable logistic regression analysis (Table 2), older age (50–60 years: adjusted OR 3.49, 95%CI 2.09—5.85 *P* < 0.001; 60–100 years: adjusted OR 4.28, 95%CI 2.21—8.17, *P* < 0.001) was still an independent predictor of seropositive syphilis. However, there were no statistical differences in gender, marital status, ART regimen, HIV viral load and level of hospital care.

**Table 2:** Factors associated with the risk of a positive serology of syphilis

Characteristic	unadjusted OR^a^	95% CI	*p*-value	adjusted OR^b^	95% CI	*p*-value
Age (years)						
(0,30]	—	—	—	—	—	
(30,40]	0.88	0.57, 1.36	0.6	0.90	0.56, 1.45	0.7
(40,50]	1.50	0.98, 2.29	0.061	1.44	0.88, 2.36	0.15
(50,60]	3.77	2.49, 5.72	< 0.001	3.49	2.09, 5.85	< 0.001
(60,100]	5.48	3.26, 9.07	< 0.001	4.28	2.21, 8.17	< 0.001
Sex						
Female	—	—	—	—	—	
Male	1.08	0.78, 1.48	0.6	0.97	0.67, 1.39	0.9
Marriage						
Divorced	—	—	—	—	—	
Married	2.24	0.69, 13.8	0.3	2.38	0.71, 14.9	0.2
Separated	2.25	0.52, 15.5	0.3	2.05	0.44, 14.6	0.4
Single	1.35	0.40, 8.38	0.7	1.81	0.52, 11.5	0.4
Widowed	3.79	1.12, 23.6	0.071	2.69	0.77, 17.0	0.2
ART						
no	—	—	—	—	—	
yes	0.92	0.55, 1.65	0.8	0.62	0.11, 11.9	0.7
ART regimen						
TLE	—	—	—	—	—	
TLD	1.36	0.96, 1.99	0.1	1.38	0.93, 2.09	0.12
Others	0.45	0.07, 1.50	0.3	0.23	0.01, 1.14	0.2
Unknown	0.86	0.41, 1.65	0.7	0.84	0.36, 1.77	0.7
Duration of ART (months)						
0 ~ 6	—	—		—	—	
6 ~ 12	1.07	0.50, 2.18	0.9	0.97	0.45, 2.02	> 0.9
12 ~ 24	1.22	0.66, 2.28	0.5	1.15	0.61, 2.19	0.7
24 ~	1.60	1.02, 2.63	0.053	1.33	0.81, 2.27	0.3
HIV viral load (copies/mL)						
< 1000	—	—	—	—	—	
≥ 1000	0.82	0.54, 1.19	0.3	1.02	0.61, 1.63	> 0.9
Hospital						
primary	—	—	—	—	—	
secondary	1.13	0.61, 1.99	0.7	1.23	0.64, 2.27	0.5
tertiary	0.92	0.66, 1.31	0.6	0.91	0.62, 1.34	0.6

*ART* antiretroviral therapy, *CI* confidence interval, *OR* odd ratio, *TLD* tenofovir + lamivudine + dolutegravir, *TLE* tenofovir + lamivudine + efavirenz

^a^Univariate logistic regression results

^b^multivariate logistic regression adjusted for age, sex, marriage, ART, ART regimen, duration of ART and type of hospital. A two-tailed *P*-value less than 0.05 was deemed to be statistically significant

## Discussion

We observed that the seroprevalence of syphilis among PLHIV was 6.8%. Older adults in this study were more likely to be seropositive for syphilis than younger adults but no statistical significance in the gender, ART status, marital status, and the level of HIV viral load and hospital care.

Our study has several strengths. First, it is the first large-scale study to determine the seroprevalence of syphilis among PLHIV in Sierra Leone. Second, its findings add to the global context of triple elimination of syphilis, HIV, and viral hepatitis B [21]. Third, it was a country-wide assessment of the seroprevalence of syphilis among PLHIV in the national referral, regional and district hospitals, and are likely representative of the epidemiologic situation of syphilis in Sierra Leone. Furthermore, our study provides data for comparison with findings of other studies in Africa and elsewhere. Finally, understanding the burden of syphilis is relevant to achieving the Sustainable Development Goals, particularly those focused on reducing communicable disease [22].

Our study has several policy and public health implications. It is encouraging that the reported prevalence of syphilis in this study is similar to the prevalence of syphilis among PLHIV reported in Ethiopia [23]. Although the prevalence of syphilis in our study may have been overestimated due to the limitations of the test kits in reacting to non-treponemal infections, studies in other African countries such as Nigeria (14.0%), Ghana (14.8%), Morocco (16.4%), Chad (14.0%), and Tanzania (9.6%) reported a higher prevalence [8–10, 24–26]. The disparities in these findings may be due to the different composition of the study populations and differences in the methods applied for the detection of syphilis or perhaps may reflect the variations in the risky behavioral practices across the African region.

Although there is no relevant local report on syphilis infection rates among PLHIV and non-HIV individuals. The higher incidence of syphilis in PLHIV compared with non-HIV individuals has been reported by others and is partly due to the shared risk behaviors associated with both infections [9, 24]. Therefore, the high prevalence of syphilis in PLHIV in our study indicates an ongoing involvement of this population in high-risk sexual behaviors, highlighting the need for continuous counseling of this group on consistent safe sex practices and routine screening for syphilis, given the clinical implications of this coinfection.

A slight male preponderance was observed, although it did not reach statistical significance (*P* = 0.6). These findings are similar to studies in Morocco and Ethiopia, but different from studies in Taiwan, Huston Texas, Brazil, Tanzania and Nigeria. We can partly explain this by the higher number of females enrolled in our study and higher number of MSM (high risk group for not only syphilis but STIs in general) enrolled in the other studies [8, 10, 23, 24, 26–28].

The study also noted that the prevalence of syphilis increases with age, a finding that contrasts with findings from Uganda, which reported high prevalence among young people [29]. Our findings can be explained by the fact that in our country, most STI and safe sex campaigns are targeted towards young people and key populations compared to the elderly, who because of the effectiveness and availability of ART, now live longer and healthier, with tendencies to practice unsafe sex [30]. In addition, we cannot rule out a role for the differences in health education and the service they received between young and old people. In addition, the decline in immunity with age might further compound the fragile immunity of PLHIV. Overall, this finding presents a unique opportunity to study the risk factors for syphilis and /or STIs in elderly PLHIV.

Syphilis seropositivity was lower in never married participants and high in previously married PLHIV with the highest prevalence seen in widowed PLHIV. This parallels studies in Ghana and Ethiopia [9, 31]. The reasons for the difference in syphilis prevalence between married and unmarried participants are unclear, but a Chinese study reported that marriage had a limited effect in reducing high-risk sexual behaviors [32]. This may explain the lower prevalence of syphilis among unmarried participants.

Despite its statistical insignificance, our study showed a higher seropositivity in ART naive clients, similar to studies in Canada and China [10, 11, 31]. Observations from some studies have implicated ART in enhancing susceptibility to syphilis infection, by suppressing proinflammatory response, while other studies have reported high risk sexual behaviors of PLHIV on ART due to low-risk perception, individual knowledge of HIV positivity, new HIV diagnosis, longer duration on ART and non-disclosure of HIV status to partner with counter findings in another study [33–41].

Finally, the seroprevalence of syphilis was higher in virally unsuppressed patients, a finding analogous to that in Tanzania [26]. Clinical stability and viral suppression may give a false sense of recovery in PLHIV which may lead them to involvement in high-risk sexual behavior [42, 43]. This calls for continuous STI and safe sex education in all PLHIV regardless of their clinical, virological or immunological success.

Our study has limitations. We did not collect clinical information related to syphilis, which made it difficult to determine the clinical presentation. The test kits used in this study did not differentiate between active or past exposure to syphilis. In addition, we only determined the syphilis seroprevalence in PLHIV, not in healthy controls. And we did not evaluate the high-risk sexual behavior of PLWH in our study. Finally, since the kit used in this study was for screening, there may be false positive reactions for non-treponemal infections such as yaws, leading to an overestimation of the burden of syphilis among PLHIV Sierra Leone. Nonetheless, we present evidence that could inform the introduction of routine screening for syphilis in PLHIV in Sierra Leone.

## Conclusion

We observed a high prevalence of syphilis in PLHIV and that older adults were more likely than young people to be seropositive for syphilis. It calls for the integration of syphilis screening, treatment and prevention in HIV services as studies have reported asymptomatic and atypical presentations which can be easily missed if routine screenings are not done.

## Data Availability

The data that support the findings of this study are available from the corresponding author Guang Yang upon reasonable request.

## Abbreviations

AIDS: Acquired immunodeficiency syndrome
ANOVA: Analysis of variance
ART: Antiretroviral therapy
EDTA: Ethylenediaminetetraacetic acid
HIV: Human immunodeficiency virus
IQR: Interquartile range
LMIC: Low- and middle-income countries
MSM: Men who have sex with men
aOR: Adjusted odds ratio
PLHIV: People living with HIV
RNA: Ribonucleic acid
STI: Sexually transmitted infections
WHO: World Health Organization
CE: European Conformity

## References

[1] Global progress report on HIV, viral hepatitis and other sexually transmitted infections. (2021). Available at: https://www.who.int/data/gho/data/themes/topics/data-on-syphilis. (Accessed 2 June 2023).

[2] Newman L, Kamb M, Hawkes S, Gomez G, Say L, Seuc A, Broutet N (2013). Global estimates of Syphilis in pregnancy and associated adverse outcomes: analysis of multinational antenatal surveillance data. PLoS Med.

[3] Tripathy DM, Gupta S, Vasudevan B (2022). Resurgence of Syphilis, the great imitator. Med J Armed Forces India.

[4] Orbe-Orihuela YC, Sánchez-Alemán MÁ, Hernández-Pliego A, Medina-García CV, Vergara-Ortega DN (2022). Syphilis as Re-emerging Disease, Antibiotic Resistance, and Vulnerable Population: global systematic review and Meta-analysis. Pathogens.

[5] Palacios R, Jimenez-Onate F, Aguilar M (2007). Impact of Syphilis Infection on HIV viral load and CD4 cell counts in HIV-infected patients. J Acquir Immune Defic Syndr.

[6] Koga I, Odawara T, Matsuda M, Sugiura W, Goto M, Nakamura T, Iwamoto A (2006). Analysis of HIV-1 sequences before and after co-infecting Syphilis. Microbes Infect.

[7] Global health sector. strategies on, respectively, HIV, viral hepatitis and sexually transmitted infections for the period 2022–2030. Available at: https://www.who.int/teams/global-hiv-hepatitis-and-stis-programmes/strategies/global-health-sector-strategies#:~:text=The%20strategies%20aim%20to%20end,WHO’s%20General%20Programme%20of%20Work. (Accessed 2 June 2023).

[8] Bourouache M, Mimouni R, Nejmeddine M, Chadli S, Benlmeliani F, Sardi J, Malmoussi M, Ouagari Z, Basbassi ME, Aghrouch M (2019). The prevalence of Syphilis in HIV-seropositive patients: a retrospective study at the regional hospital in Agadir, Morocco. Pan Afr Med J.

[9] Mamoojee Y, Tan G, Gittins S, Sarfo S, Stephenson L, Carrington D, Bedu-Addo G, Phillips R, Appiah LT, Chadwick D (2012). Diagnosis of treponemal co-infection in HIV-infected West Africans. Trop Med Int Health.

[10] Lee NY, Chen YC, Liu HY, Li CY, Li CW, Ko WC, Ko NY (2020). Increased repeat Syphilis among HIV-infected patients: a nationwide population-based cohort study in Taiwan. Med (Baltim).

[11] Lang R, Read R, Krentz HB, Ramazani S, Peng M, Gratrix J, Gill MJ (2018). Increasing incidence of Syphilis among patients engaged in HIV care in Alberta, Canada: a retrospective clinic-based cohort study. BMC Infect Dis.

[12] Sarkodie F, Owusu-Dabo E, Hassall O, Bates I, Bygbjerg IC, Ullum H (2016). Recall of symptoms and treatment of Syphilis and yaws by healthy blood donors screening positive for Syphilis in Kumasi, Ghana. Int J Infect Dis.

[13] Sierra Leone Demographic Health Survey (SLDHS). 2019. Available at: https://dhsprogram.com/pubs/pdf/PR122/PR122.pdf. (Accessed on 3 June 2023).

[14] UNAIDS Country progress report - Sierra Leone. Available at: https://www.unaids.org/sites/default/files/country/documents/SLE_2020_countryreport.pdf. (Accessed 2 June 2023).

[15] Djibo DA, Sahr F, McCutchan JA, Jain S, Araneta MRG, Brodine SK, Shaffer RA (2017). Prevalence and risk factors for human immunodeficiency virus (HIV) and Syphilis Infections among Military Personnel in Sierra Leone. Curr HIV Res.

[16] Yambasu EE, Reid A, Owiti P, Manzi M, Murray MJS, Edwin AK (2018). Hidden dangers-prevalence of blood borne pathogens, Hepatitis B, C, HIV and Syphilis, among blood donors in Sierra Leone in 2016: opportunities for improvement: a retrospective, cross-sectional study. Pan Afr Med J.

[17] Yendewa GA, Lakoh S, Yendewa SA, Bangura K, Lawrence H, Patiño L, Jiba DF, Vandy AO, Murray MJS, Massaquoi SP, Deen GF, Sahr F, Hoffmann CJ, Jacobson JM, Poveda E, Aguilera A, Salata RA (2021). Prevalence of Hepatitis B surface antigen and serological markers of other endemic Infections in HIV-infected children, adolescents and pregnant women in Sierra Leone: a cross-sectional study. Int J Infect Dis.

[18] GoSL SLMTDP Development-Documents-National-Development-Plan-2019-23-47099. 2018. Available online: https://www.imf.org/en/Publications/CR/Issues/2019/07/09/Sierra-Leone-Economic-Development-DocumentsNational-Development-Plan-2019-23-47099. [(Accessed on 14 January 2022)].

[19] SSL. 2015: Sierra Leone Population Census. Available online: https://www.statistics.sl/images/StatisticsSL/Documents/Census/2015/sl_2015_phc_thematic_report_on_pop_structure_and_pop_distribution.pdf. (Accessed on 14 January 2022).

[20] MICS 2017: Sierra leone multiple indicator cluster survey. 2017. Available online: https://www.statistics.sl/images/StatisticsSL/Documents/sierra_leone_mics6_2017_report.pdf. (Accessed on 14 January 2022).

[21] Global health sector. strategies on, respectively, HIV, viral hepatitis and sexually transmitted infections for the period 2022–2030. Available at: https://iris.who.int/bitstream/handle/10665/360348/9789240053779-eng.pdf?sequence=1. Accessed 16 October 2023).

[22] United Nations Sustainable Development Goals. [(Accessed on 16 October 2023)]. Available online: https://sdgs.un.org/goals.

[23] Shimelis T, Lemma K, Ambachew H (2015). Syphilis among people with HIV Infection in southern Ethiopia: sero-prevalence and risk factors. BMC Infect Dis.

[24] Uneke C, Ogbu O, Alo M, Obaji A (2006). Syphilis serology in HIV-positive and HIV-negative Nigerians: The public health significance.

[25] Adawaye C, Souleymane AO, Fouda AA, Djarma O, Cournil A, Tuaillon E, Mennechet FJD (2021). Syphilis diagnosis and serological response to Benzathine Penicillin G among patients attending HIV clinics in N’Djaména, Chad. Int J Infect Dis.

[26] Haule A, Msemwa B, Mgaya E, Masikini P, Kalluvya S (2020). Prevalence of Syphilis, neurosyphilis and associated factors in a cross-sectional analysis of HIV infected patients attending Bugando Medical Centre, Mwanza, Tanzania. BMC Public Health.

[27] Yang B, Hallmark CJ, Huang JS, Wolverton ML, McNeese-Ward M, Arafat RR (2013). Characteristics and risk of syphilis diagnosis among HIV-infected male cohort: a population-based study in Houston. Texas. Sex Transm Dis.

[28] Adolf R, Bercht F, Aronis ML, Lunardi LW, Schechter M, Sprinz E (2012). Prevalence and risk factors associated with Syphilis in a cohort of HIV positive individuals in Brazil. AIDS Care.

[29] Mboowa G, Inda D (2015). Seroprevalence of Syphilis among Human Immunodeficiency Virus Positive Individuals Attending Immune Suppressed Syndrome Clinic at International Hospital Kampala, Uganda. Int STD Res Rev.

[30] Nakagawa F, May M, Phillips A. Life expectancy living with HIV: recent estimates and future implications. Current Opinion in Infectious Diseases. 2013;26(1):p 17–25. | 10.1097/QCO.0b013e32835ba6b1. 10.1097/QCO.0b013e32835ba6b1 23221765

[31] Tura JB, Ayalew J, Moreda AB (2023). Prevalence of Syphilis and associated factors among female sex workers in Ethiopia: findings from a multilevel analysis of a national bio-behavioral survey. BMC Public Health.

[32] Guo YL, Zhou JB, Hao C, Huan XP, Shi TP, Wang JT, Zhen S, Yin YP (2013). [Comparative analysis on both high risk behaviours, Infection of HIV and Syphilis between married and unmarried men who have sex with men]. Zhonghua Liu Xing Bing Xue Za Zhi.

[33] Hu Q, Xu J, Zou H (2014). Risk factors associated with prevalent and incident Syphilis among an HIV-infected cohort in Northeast China. BMC Infect Dis.

[34] Rekart ML, Ndifon W, Brunham RC, Dushoff J, Park SW, Rawat S, Cameron CE (2017). A double-edged sword: does highly active antiretroviral therapy contribute to Syphilis incidence by impairing immunity to Treponema pallidum?. Sex Transm Infect.

[35] Crepaz N, Marks G, Liau A (2009). Prevalence of unprotected anal intercourse among HIV-diagnosed MSM in the United States: a meta-analysis. AIDS.

[36] Stephenson JM, Imrie J, Davis MMD (2003). Is use of antiretroviral therapy among homosexual men associated with increased risk of transmission of HIV Infection?. Sex Transm Infect.

[37] Remien RH, Halkitis PN, O’Leary A (2005). Risk perception and sexual risk behaviors among HIV-positive men on antiretroviral therapy. AIDS Behav.

[38] Chen SC, Wang ST, Chen KT, Yan TR, Tang LH, Lin CC, Yen SF. Analysis of the influence of therapy and viral suppression on high-risk sexual behaviour and sexually transmitted infections among patients infected with human immunodeficiency virus in Taiwan. Clin Microbiol Infect. 2006;12(7):660–5. 10.1111/j.1469-0691.2006.01473.x 16774563

[39] Vu TMT, Boggiano VL, Tran BX, Nguyen LH, Tran TT, Latkin CA, Ho CSH, Ho RCM (2018). Sexual risk behaviors of patients with HIV/AIDS over the Course of Antiretroviral Treatment in Northern Vietnam. Int J Environ Res Public Health.

[40] Luchters S, Sarna A, Geibel S, Chersich MF, Munyao P, Kaai S, Mandaliya KN, Shikely KS, Rutenberg N, Temmerman M (2008). Safer sexual behaviors after 12 months of antiretroviral treatment in Mombasa, Kenya: a prospective cohort. AIDS Patient Care STDS.

[41] Jean K, Gagillard D, Moh R (2014). Decrease in sexual risk behaviours after early initiation of antiretroviral therapy: a 24-month prospective study in Côte d’Ivoire. J Int AIDS Soc.

[42] Hasse B, Ledergerber B, Hirschel B, Vernazza P, Glass TR, Jeannin A, Evison JM, Elzi L, Cavassini M, Bernasconi E, Nicca D, Weber R, Swiss HIV (2010). Cohort study. Frequency and determinants of unprotected sex among HIV-infected persons: the Swiss HIV cohort study. Clin Infect Dis.

[43] Dukers NH, Goudsmit J, de Wit JB, Prins M, Weverling GJ, Coutinho RA (2001). Sexual risk behaviour relates to the virological and immunological improvements during highly active antiretroviral therapy in HIV-1 infection. AIDS.

